# Patients in palliative care—Development of a predictive model for anxiety using routine data

**DOI:** 10.1371/journal.pone.0179415

**Published:** 2017-08-03

**Authors:** Sonja Hofmann, Stephanie Hess, Carsten Klein, Gabriele Lindena, Lukas Radbruch, Christoph Ostgathe

**Affiliations:** 1 Department of Palliative Medicine, Universitätsklinikum Erlangen, CCC Erlangen-EMN, Friedrich- Alexander-Universität Erlangen-Nürnberg, Erlangen, Germany; 2 Department of Anaesthesiology, Universitätsklinikum Erlangen, CCC Erlangen-EMN, Friedrich-Alexander-Universität Erlangen-Nürnberg, Erlangen, Germany; 3 Clinical Analysis, Research and Application (CLARA), Kleinmachnow, Germany; 4 Department of Palliative Medicine, University Hospital of Bonn, Bonn, Germany; Nanjing Normal University, CHINA

## Abstract

**Introduction:**

Anxiety is one of the most common psychological symptoms in patients in a palliative care situation. This study aims to develop a predictive model for anxiety using data from the standard documentation routine.

**Methods:**

Data sets of palliative care patients collected by the German quality management benchmarking system called Hospice and Palliative Care Evaluation (HOPE) from 2007 to 2011 were randomly divided into a training set containing two-thirds of the data and a test set with the remaining one-third. We dichotomized anxiety levels, proxy rated by medical staff using the validated HOPE Symptom and Problem Checklist, into two groups with no or mild anxiety versus moderate or severe anxiety. Using the training set, a multivariable logistic regression model was developed by backward stepwise selection. Predictive accuracy was evaluated by the area under the receiver operating characteristic curve (AUC) based on the test set.

**Results:**

An analysis of 9924 data sets suggests a predictive model for anxiety in patients receiving palliative care which contains gender, age, ECOG, living situation, pain, nausea, dyspnea, loss of appetite, tiredness, need for assistance with activities of daily living, problems with organization of care, medication with sedatives/anxiolytics, antidepressants, antihypertensive drugs, laxatives, and antibiotics. It results in a fair predictive value (AUC = 0.72).

**Conclusions:**

Routinely collected data providing individual-, disease- and therapy-related information contain valuable information that is useful for the prediction of anxiety risks in patients receiving palliative care. These findings could thus be advantageous for providing appropriate support for patients in palliative care settings and should receive special attention in future research.

## Introduction

Psychological symptoms in patients with terminal diseases are very frequent [1–5]. Anxiety is one of the most common psychological symptoms in patients in palliative care settings [2]. In a meta-analysis of 24 studies on mood disorders in patients with terminal diseases, a pooled prevalence for anxiety disorders of 9.8% (Range: 6.8%– 13.2%) was reported [2]. This high prevalence and the evidence that psychological symptoms can lower quality of life should lead to increased sensitivity toward psychological burdens among clinicians who care for such patients [6–8].

In addition to the description of the prevalence of symptom burden in patients suffering from a terminal disease, many studies focus on interrelations of symptoms [6]. For anxiety, several associations with other symptoms in patients with advanced cancer or in palliative care settings can be reported from previous research: Delgado-Guay et al. [7] and Oechsle et al. [8] describe associations of anxiety with physical symptom burden. In particular, there are findings of interrelations with pain [7, 9, 10], fatigue [7], sleep disturbance [6], nausea [7, 9], dyspnea [7, 9, 11], and cardiac arrhythmias [9]. Although there are findings indicating no relationship between anxiety and socio-demographic or personal data [12], later analyses showed associations between anxiety and younger age [6] as well as being female [6, 13, 14]. Further findings, such as associations of anxiety with use of medications [13], acceptance [15], and belief in an afterlife [13], are reported elsewhere [11, 16, 17].

Due to the small sample sizes and the heterogeneity of many studies, it is still challenging to find reliable predictors for anxiety in terminally ill patients [6].

### Objectives of the study

The objectives of the study are (i) to describe sample characteristics of routine data collected in palliative care settings in Germany, (ii) to explore differences between data sets of patients with high anxiety scores and those with low anxiety scores, (iii) to discover possible predictors of anxiety, and (iv) to find and test a predictive model based on routine data.

## Materials and methods

### Study material and data sets

For this study we used anonymized data of adult patients from palliative care services in Germany. Data were collected using the Hospice and Palliative Care Evaluation (HOPE), a long-term, web-based quality management benchmarking system for palliative care, which is well-established in Germany. HOPE was developed as a core data set by experts from a range of professions from the German Association for Palliative Medicine, the German Hospice and Palliative Care Association, and the German Association for Cancer [18–20]. It is composed of different modules and can be used by inpatient or outpatient hospice and palliative care services. To collect information about personal, disease-related and therapy-related data, HOPE includes a core data set (see Supporting Information S1, S2 and S3 Figs), which is assessed by medical staff (e.g. physicians, nurses). To assess the individual symptom burden of patients with terminal diseases, it contains a validated symptom and problem checklist (HOPE-SP-CL) [21]. Using a four-point grading scale (none, mild, moderate, severe), HOPE-SP-CL assesses individual physical (pain, nausea, vomiting, dyspnea, constipation, weakness, loss of appetite, tiredness), psychological (feeling depressed, anxiety, tension, disorientation/confusion), social (organization of care, overburdening of family) and nursing (wound care, assistance with activities of daily living [ADLs]) burden at least once on admission to the service and once on the day of discharge or death of the patient. HOPE is not used to diagnose mental disorders. Rather, it is used to assess the current suffering of patients. In particular, mental symptoms such as anxiety and feeling depressed can be very painful if they occur in the course of a serious, fatal illness, even if the criteria of a mental disorder are not met. Data sets are extracted from the standardized documentation systems of the participating services each year for a period of three months (March 15 –June 14) to maintain a continuous benchmarking process. Data sets are centrally merged and processed and are then fed back to the services in benchmark format through a predefined and programmed interface. For this study we used data sets of patients in different inpatient and outpatient institutions (palliative care units, hospital support teams, oncology wards, hospices, palliative home care services) in Germany from 2007 to 2011 [20].

### Ethics and data protection

No additional data collection was necessary and we did not make any changes to the care or treatment of patients to perform this study. The data sets used were completely depersonalized. The institutional ethics review board of Friedrich-Alexander-Universität Erlangen-Nürnberg was formally approached and did not have any ethical or legal concerns about this study.

### Data analysis and model development

IBM SPSS Statistics 21.0 for Windows (IBM Corporation, Armonk, NY) was used for statistical evaluation.

In order to characterize the sample, descriptive analyses were performed. Symptom scores of anxiety were dichotomized into none or mild intensity versus moderate or severe intensity [22, 23].

The data set was divided into a training set consisting of two thirds of the data and a test set consisting of one third of the data. On the basis of the training set, a broad range of variables (e.g. demographic, medical and psychosocial items) of the routine data were used to search for bivariate interrelations with the command variable. Afterwards, variables showing a significant association (using a significance level of p < .25) with the dichotomized ratings of anxiety were considered for the development of a multivariable logistic regression model, which was generated using a backward stepwise selection. Due to the high intercorrelations with the target variable, other items related to psychological burden such as feeling depressed, tension and disorientation/confusion were not included in the analysis.

On the basis of the test set, which comprises the remaining one third of data, predictive accuracy of the final model was evaluated by the area under the receiver operating characteristic curve (AUC) corresponding with the c statistic. An AUC of .80 or higher indicates good sensitivity and specificity, whereas fair sensitivity and specificity is described between AUCs of .70 and .80. Positive and negative predictive values were also calculated [24].

## Results

### Sample characteristics

**Fig 1:**
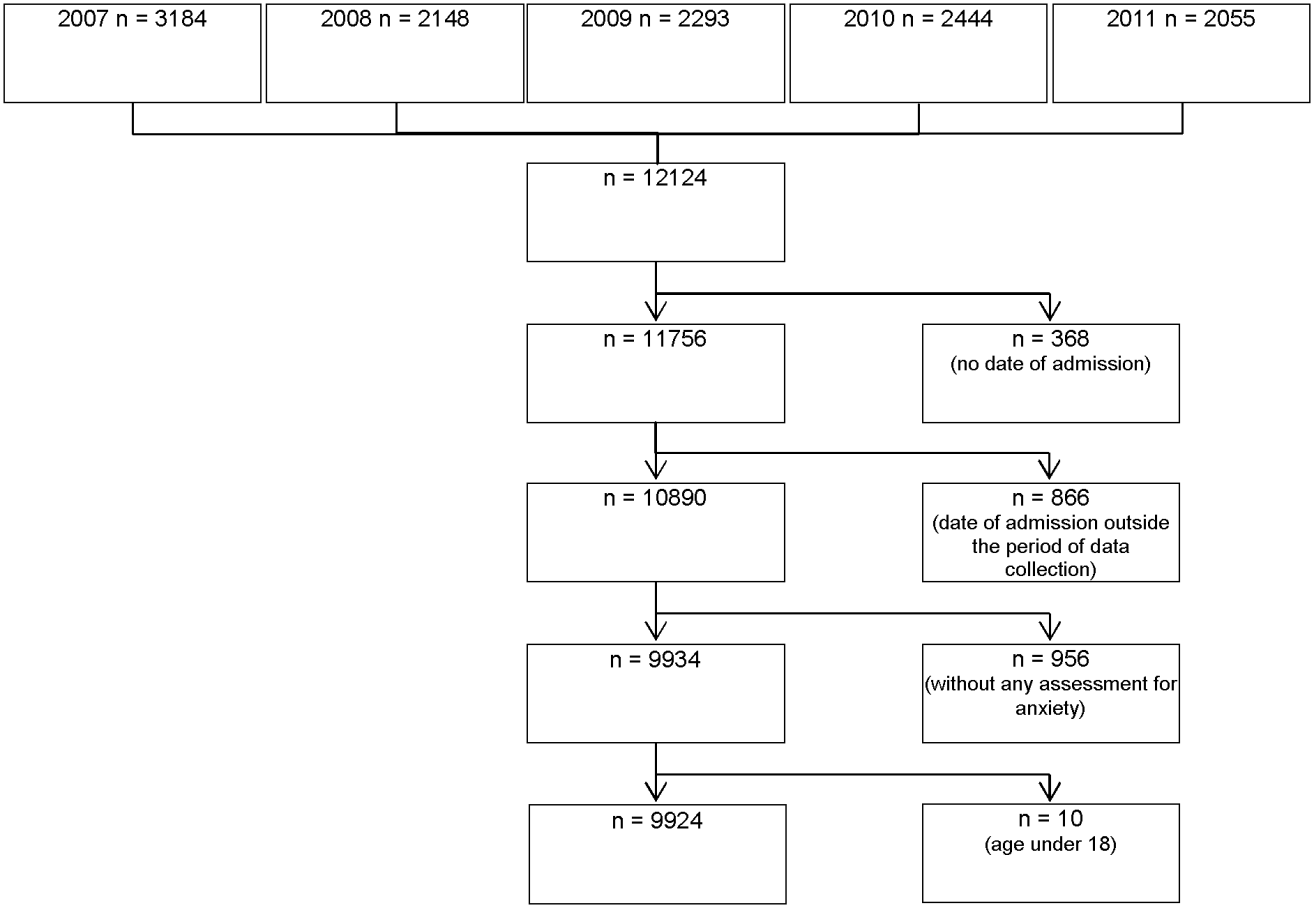
Flow of study participants.

Overall, 12124 data sets of patients transferred to HOPE by the participating inpatient or outpatient palliative care services during the period under review in the years 2007 to 2011 were used in this study (2007 n = 3184, 2008 n = 2148, 2009 n = 2293, 2010 n = 2444, 2011 n = 2055). The following data sets were excluded from further analyses: 368 data sets due to missing date of admission to the palliative care service, 866 data sets because date of admission to the palliative care service was outside the annual three month period of data collection, 956 data sets without any assessment for anxiety and 10 data sets due to a documented age under 18. Fig 1 shows this selection of the data sets. Comparisons between participants (n = 9924) and non-participants (n = 2200) yielded no significant differences in age, gender, performance status (ECOG) or palliative care services at time of admission.

Table 1 shows the characteristics of the study sample of 9924 patients who were assessed with HOPE at time of admission to a palliative care service (see Supporting Information S1 File). Just over half were female (51.9%) and the average age was 68.8 (Range: 19.3–109.4; SD = 12.6). The majority of patients showed a low performance status (33.7% ECOG 3, 37.5% ECOG 4) at the time of admission. For 36.2% of the population under review, no level of nursing care support had been approved by the health insurance providers. (Note: According to the German long-term care legislation in the period under review, patients may receive reimbursement for nursing care. In order for this to occur, they have to apply for a level of nursing care support. Reimbursement is granted for one of four levels of nursing care support (level 1: necessary support for a minimum of two activities in fields of personal hygiene, eating and mobility (at least once a day), housekeeping (at least several times a week) with an overall duration of care at least 90 min a day; level 2: necessary support at least three times a day with an overall (average) duration of 3 h a day; level 3: ongoing need for support (including at night), average duration of at least 5 h a day; level 3+: special cases of hardship with an excessive high need for support, and need for support for artificial home ventilation). Compared to other variables, level of nursing care support showed by far the highest rate of missing values (14.7%) and was therefore excluded from further calculations. Most of the patients had a cancer diagnosis (89.2%) and lived with family members (66.4%) or alone (21.7%). Advance directives were formulated by approximately one third (32.2%) of the study population. Two thirds were patients from a specialized inpatient palliative care unit (60.4%); 24.1% were supported by outpatient services. 38.3% of patients included in this study were assessed with moderate or severe anxiety levels.

**Table 1:** Sample characteristics (N = 9924).

Item	Categories	%	(n)
**Age**	≤ 58.2	19.7	(1959)
	58.21–67.21	19.7	(1959)
	67.22–72.80	19.7	(1955)
	72.81–79.69	19.7	(1952)
	≥ 79.70	21.0	(2082)
	Missing values	.2	(17)
**Gender**	Male	47.3	(4694)
	Female	51.9	(5149)
	Missing values	.8	(81)
**Performance status (ECOG)**	ECOG 0	2.1	(206)
	ECOG 1	6.4	(638)
	ECOG 2	16.5	(1634)
	ECOG 3	33.7	(3347)
	ECOG 4	37.5	(3726)
	Missing values	3.8	(373)
**Living situation**	Alone	21.7	(2157)
	Nursing home	4.8	(476)
	With family members	66.4	(6590)
	Other	2.8	(277)
	Missing values	4.3	(424)
**Palliative care service**	Palliative care unit	60.9	(6039)
	Other ward	2.5	(246)
	Hospice	10.8	(1076)
	Outpatient service	23.3	(2317)
	Missing values	2.5	(246)
**Level of nursing care support** (according to German long-term legislation)	None	36.2	(3595)
	1 (at least 90 min a day in assistance with body care, feeding, mobilization, and housing)	13.9	(1378)
	2 (at least 3 h per day)	11.3	(1126)
	3 (at least 5 h per day)	3.1	(306)
	3+ (at least 5 h per day and additional need in assistance with artificial home ventilation)	.3	(32)
	Classification applied	20.8	(2061)
	Missing values	14.4	(1426)
**Advance directives**	Yes	32.2	(3198)
	Not known/missing values	67.8	(6726)
**Type of disease**	Cancer	89.2	(8848)
	Non-cancer	6.7	(665)
	Unclear	2.6	(254)
	Missing values	1.6	(157)
**Brain metastases**	Yes	14.4	(1425)
	Not known/missing values	85.6	(8499)
**Anxiety**	None or mild	61.7	(6127)
	Moderate or severe	38.3	(3797)
**Feeling depressed**	None or mild	68.3	(6563)
	Moderate or severe	31.7	(3042)
**Tension**	None or mild	57.3	(5571)
	Moderate or severe	42.7	(4154)
**Disorientation/confusion**	None or mild	82.0	(7911)
	Moderate or severe	18.0	(1740)

### Development and evaluation of the predictive model

For 35 variables assessed by HOPE, bivariate interrelations with anxiety were calculated on the basis of the training set. The results of these analyses are shown in Table 2. The following variables showed no significant association with anxiety and were therefore not included in further calculations: type of service, advance directives, type of disease, brain metastases, medication with opioids (WHO step 2), and medication with diuretics. Twenty-nine variables showing a significant association with anxiety were put into a backward stepwise logistic regression model as candidate predictor variables. Table 3 shows the final model comprising those variables, whose influence on the target variable persists when controlling for this wide range of variables. The following 15 variables remained in the final logistic regression model: gender, age, ECOG, living situation, pain, nausea, dyspnea, loss of appetite, tiredness, assistance with ADL, organization of care, medication with sedatives/anxiolytics, medication with antidepressants, cardiac medication, and medication with antibiotics.

**Table 2:** Results of univariate logistic regression models investigating associated factors with anxiety (training set) (N = 6581).

Item	Categories	Odds ratio (95% CI)	p	% (n) missing
**Age**	≤ 58.2	Reference	< .0001	.2% (11)
	58.21–67.21	0.82 (0.70, 0.95)		
	67.22–72.80	0.74 (0.63, 0.87)		
	72.81–79.69	0.64 (0.55, 0.75)		
	≥ 79.70	0.51 (0.43, 0.59)		
**Gender**	Male	Reference	< .0001	.9% (57)
	Female	1.31 (1.18, 1.44)		
**Performance status (ECOG)**	ECOG 0	Reference	< .0001	3.6% (233)
	ECOG 1	1.29 (0.85, 1.98)		
	ECOG 2	1.42 (0.96, 2.11)		
	ECOG 3	1.78 (1.21, 2.62)		
	ECOG 4	2.09 (1.43, 3.07)		
**Living situation**	Alone	Reference	< .0001	4.2% (271)
	Nursing home	0.96 (0.69, 1.33)		
	With family members	0.91 (0.77, 1.07)		
	Other	0.99 (0.88, 1.12)		
**Palliative care service**	Palliative care unit	Reference	.7280	2.5% (160)
	Other ward	0.82 (0.57, 1.16)		
	Hospice	0.87 (0.73, 1.04)		
	Outpatient service	0.94 (0.83, 1.07)		
**Advance directives**	Not known	Reference	.5713	(0)
	Yes	0.97 (0.87, 1.08)		
**Type of disease**	Cancer	Reference	.6252	4.1% (266)
	Non-cancer	1.05 (0.86, 1.28)		
**Brain metastases**	No	Reference	.8889	(0)
	Yes	1.12 (0.97, 1.30)		
**Pain**	None	Reference	< .0001	.7% (44)
	Mild	1.60 (1.37, 1.87)		
	Moderate	2.03 (1.75, 2.36)		
	Severe	2.95 (2.53, 3.44)		
**Nausea**	None	Reference	< .0001	.8% (55)
	Mild	1.32 (1.17, 1.50)		
	Moderate	2.09 (1.81, 2.41)		
	Severe	2.16 (1.82, 2.56)		
**Vomiting**	None	Reference	< .0001	1.0% (62)
	Mild	1.30 (1.12, 1.51)		
	Moderate	1.69 (1.42, 2.00)		
	Severe	1.86 (1.52, 2.28)		
**Dyspnea**	None	Reference	< .0001	.7% (45)
	Mild	1.50 (1.31, 1.71)		
	Moderate	1.80 (1.56, 2.07)		
	Severe	3.35 (2.88, 3.90)		
**Constipation**	None	Reference	< .0001	2.9% (192)
	Mild	1.24 (1.09, 1.41)		
	Moderate	1.52 (1.32, 1.74)		
	Severe	1.05 (1.58, 2.18)		
**Weakness**	None	Reference	< .0001	1.2% (79)
	Mild	1.54 (0.99, 2.40)		
	Moderate	2.21 (1.45, 3.36)		
	Severe	3.67 (2.43, 5.55)		
**Loss of appetite**	None	Reference	< .0001	2.4% (159)
	Mild	1.35 (1.11, 1.65)		
	Moderate	1.98 (1.65, 2.38)		
	Severe	2.53 (2.13, 3.01)		
**Tiredness**	None	Reference	< .0001	1.7% (113)
	Mild	1.29 (1.02, 1.63)		
	Moderate	1.80 (1.44, 2.25)		
	Severe	2.50 (2.01, 3.13)		
**Wound care**	None	Reference	< .0001	4.1% (266)
	Mild	1.05 (0.91, 1.22)		
	Moderate	1.47 (1.25, 1.72)		
	Severe	1.64 (1.36, 1.99)		
**Assistance with ADL**	None	Reference	< .0001	1.9% (125)
	Mild	1.24 (0.97, 1.59)		
	Moderate	1.49 (1.19, 1.88)		
	Severe	2.19 (1.76, 2.71)		
**Organization of care**	None	Reference	< .0001	4.4% (284)
	Mild	1.30 (1.12, 1.51)		
	Moderate	1.90 (1.66, 2.19)		
	Severe	2.55 (2.22, 3.94)		
**Medication with non-opioids**	No	Reference	.1099	(0)
	Yes	1.09 (0.98, 1.20)		
**Medication with opioids WHO step 2**	No	Reference	.5703	(0)
	Yes	0.95 (0.80, 1.14)		
**Medication with opioids WHO step 3**	No	Reference	< .0001	(0)
	Yes	1.48 (1.34, 1.64)		
**Medication with co-analgesics**	No	Reference	< .0001	(0)
	Yes	1.34 (1.19, 1.51)		
**Medication with corticosteroids**	No	Reference	.0021	(0)
	Yes	1.19 (1.06, 1.32)		
**Medication with sedatives/anxiolytics**	No	Reference	< .0001	(0)
	Yes	2.08 (1.86, 2.32)		
**Medication with antidepressants**	No	Reference	< .0001	(0)
	Yes	1.81 (1.60, 2.04)		
**Medication with cardiacs**	No	Reference	.0001	(0)
	Yes	0.81 (0.72, 0.90)		
**Medication with antiemetics**	No	Reference	< .0001	(0)
	Yes	1.26 (1.13, 1.40)		
**Medication with neuroleptics**	No	Reference	.0057	(0)
	Yes	1.26 (1.07, 1.47)		
**Medication with gastric protection**	No	Reference	.2235	(0)
	Yes	1.07 (0.96, 1.18)		
**Medication with laxatives**	No	Reference	.0026	(0)
	Yes	1.17 (1.06, 1.30)		
**Medication with antibiotics**	No	Reference	.0080	(0)
	Yes	1.21 (1.05, 1.39)		
**Medication with diuretics**	No	Reference	.9002	(0)
	Yes	1.01 (0.90, 1.13)		
**Medication with other drugs**	No	Reference	.0376	(0)
	Yes	0.88 (0.78, 0.99)		
**Group of diagnosis**	MN^a^ of digestive organs	Reference	.0004	1.6% (105)
	MN of respiratory and intrathoracic organs	1.23 (1.05, 1.44)		
	MN of breast	1.30 (1.08, 1.56)		
	MN of female genital organs	1.26 (1.01, 1.58)		
	MN of male genital organs	0.77 (0.60, 0.99)		
	MN of urinary tract	0.79 (0.61, 1.02)		
	MN of lymphoid, hematopoietic, and related tissue	1.16 (0.89, 1.50)		
	MN of cancer of unknown primary origin	1.28 (1.00, 1.64)		
	MN of lip, oral cavity, and pharynx	1.22 (0.89, 1.69)		
	MN of eye, brain, and other parts of the CNS^b^	0.75 (0.54, 1.05)		
	MN of mesothelial and soft tissue	1.46 (0.99, 2.14)		
	MN of skin	0.83 (0.55, 1.25)		
	Other MN	1.21 (0.76, 1.91)		
	Diseases of the circulatory system	1.07 (0.73, 1.57)		
	Diseases of the nervous system	0.94 (0.58, 1.54)		
	Diseases of the respiratory system	1.55 (0.89, 2.70)		
	Diseases of the digestive system	1.47 (0.83, 2.61)		
	Other non-malignant disorders, symptoms and injuries	1.22 (0.86, 1.74)		
	Mental and behavioral disorders	0.90 (0.40, 2.02)		
	Other diseases (e.g., malignant / benign)	1.23 (0.88, 1.72)		

^a^Malignant neoplasms (MN)

^b^Central nervous system (CNS)

**Table 3:** Final multivariable logistic regression model (training set) (N = 6518) (missing values 10.7% (1060)).

Item	Categories	Regression coefficients β	Odds ratio (95% CI)	p
**Age**	≤ 58.2		Reference	< .0001
	58.21–67.21	-.186	0.83 (0.69, 1.00)	
	67.22–72.80	-.260	0.77 (0.64, 0.93)	
	72.81–79.69	-.418	0.66 (0.54, 0.80)	
	≥ 79.70	-.635	0.53 (0.44, 0.65)	
**Gender**	Male		Reference	< .0001
	Female	.312	1.36 (1.21, 1.55)	
**Performance status (ECOG)**	ECOG 0		Reference	.0501
	ECOG 1	.277	1.32 (0.80, 2.19)	
	ECOG 2	.202	1.22 (0.76, 1.96)	
	ECOG 3	-.005	1.00 (0.62, 1.59)	
	ECOG 4	-.107	0.90 (0.56, 1.44)	
**Living situation**	Alone		Reference	< .0001
	Nursing home	.307	1.36 (1.00, 1.84)	
	With family members	.295	1.34 (1.15, 1.56)	
	Other	.814	2.26 (1.55, 3.28)	
**Pain**	None		Reference	< .0001
	Mild	.337	1.40 (1.17, 1.68)	
	Moderate	.499	1.65 (1.38, 1.97)	
	Severe	.804	2.23 (1.86, 2.69)	
**Nausea**	None		Reference	< .0001
	Mild	.089	1.09 (0.94, 1.27)	
	Moderate	.463	1.59 (1.33, 1.90)	
	Severe	.432	1.54 (1.24, 1.91)	
**Dyspnea**	None		Reference	< .0001
	Mild	.232	1.26 (1.08, 1.47)	
	Moderate	.461	1.59 (1.34, 1.88)	
	Severe	1.114	3.05 (2.55, 3.64)	
**Loss of appetite**	None		Reference	.0027
	Mild	.179	1.20 (0.95, 1.51)	
	Moderate	.367	1.44 (1.15, 1.81)	
	Severe	.392	1.48 (1.18, 1.86)	
**Tiredness**	None		Reference	.0013
	Mild	.035	1.04 (0.78, 1.37)	
	Moderate	.061	1.06 (0.81, 1.40)	
	Severe	.330	1.39 (1.05, 1.85)	
**Assistance with ADL**	None		Reference	.0127
	Mild	-.031	0.97 (0.72, 1.30)	
	Moderate	.000	1.00 (0.74, 1.35)	
	Severe	.267	1.31 (0.96, 1.78)	
**Organization of care**	None		Reference	< .0001
	Mild	.173	1.19 (1.00, 1.42)	
	Moderate	.565	1.76 (1.49, 2.08)	
	Severe	.727	2.07 (1.74, 2.46)	
**Medication with sedatives/anxiolytics**	No		Reference	< .0001
	Yes	.532	1.76 (1.52, 2.04)	
**Medication with antidepressants**	No		Reference	< .0001
	Yes	.564	2.82 (2.42, 3.27)	
**Medication with cardiacs**	No		Reference	.0012
	Yes	-.233	0.80 (0.70, 0.92)	
**Medication with antibiotics**	No		Reference	.0337
	Yes	.185	1.20 (1.01, 1.43)	
**Intercept**		-2.658		

**Fig 2:**
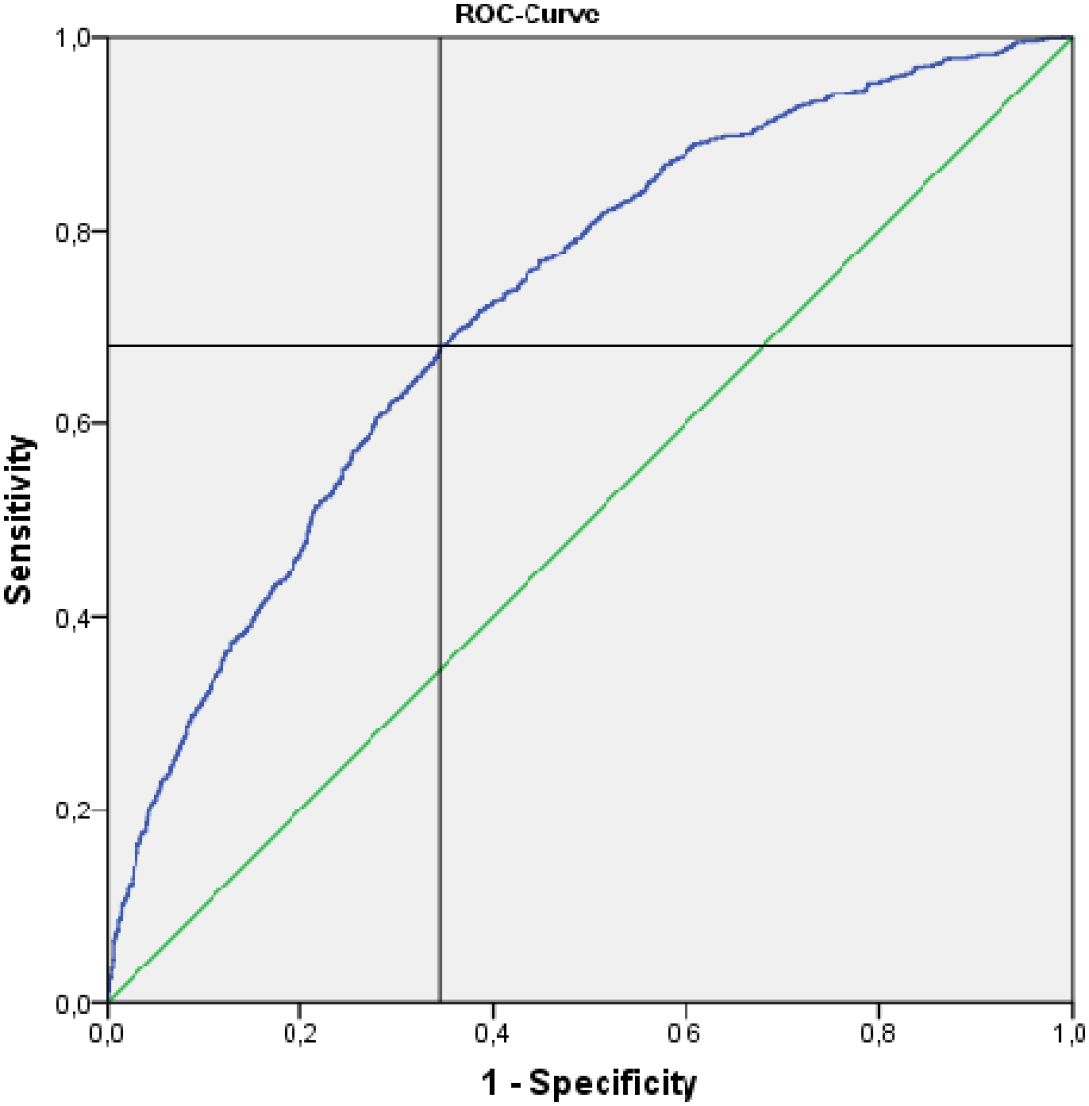
ROC curve of the final multivariable regression model. AUC (95% CI) 0.723 (0.704, 0.742). Optimal cut-off score > 0.3714649.

The model was evaluated by the test set, which consists of one third of the whole data set and showed a fair predictive value (AUC = 0.72). Fig 2 shows the ROC curve. The optimal cut-off score of our model, which maximizes sensitivity and specificity, was found to be ≥ 0.3714649. Sensitivity was 0.68 and specificity 0.66. Under consideration of the prevalence of anxiety in the test set, the negative predictive value was 0.70 and the positive predictive value was 0.61.

We also considered the impact of missing data. In most variables there is only a small proportion of missing values of 5% or less. Due to a conspicuously high level of missing data (14.4%), the item level of nursing care support was excluded from further analyses. Nevertheless, the final model shows 10.7% missing values. Since we could not find any significant difference between data sets excluded from further analyses due to missing values and those included in the final model, we assumed missing data were most likely caused by random rather than by systematic bias.

## Discussion

To the best of our knowledge the present study is the first in which such a broad range of different variables from routine data was used to find a predictive model for anxiety in patients with terminal diseases on the basis of a data set of this size which was collected nationwide.

Regarding predictors of anxiety in patients in palliative care settings, the present study yielded several results. Like previous findings, it confirms interrelations between physical symptom burden and anxiety [7–10]. For all aspects of physical burden assessed by HOPE, calculations showed highly significant bivariate associations with anxiety. Therefore, higher physical symptom burden is more likely to be accompanied by higher anxiety levels. However, despite these significant interrelations, only symptom burden from pain, nausea, dyspnea, loss of appetite, and tiredness remained in the final multivariable model after backward stepwise selection. This indicates that the influences of those variables showing a significant bivariate connection with anxiety but having been removed by backward stepwise selection were exceeded by the stronger influences of remaining variables.

In particular, our findings concerning the predictive value of dyspnea are also consistent with previous research [11]. In recent years, the linkage of anxiety and dyspnea in terminally ill patients has drawn more attention to the treatment of dyspnea with anxiolytics and with psychological interventions such as relaxation/imagery with breathing retraining in addition to treatment with opioids [25–27]. Furthermore, this connection emphasizes the importance of awareness while supporting and treating patients who are suffering from physical symptoms.

Although there are different indications concerning a linkage between age and anxiety [6, 13], our findings affirm that younger age is more likely to be connected to higher anxiety levels [6]. Additionally, our data also showed a correlation of gender of patients in palliative care settings with anxiety and thus confirmed previous findings of higher anxiety levels in women with advanced cancer or terminal diseases than in men [6, 13, 14].

Furthermore, our data underpins some findings of a study by Kolva et al. [13]. A difference in anxiety levels between inpatients and outpatients was reported in this study but this variable showed no significant interrelation in a multiple regression analysis. This is also evident in comparison with the present data analysis, where no significant influence of inpatient and outpatient palliative care services on patients’ anxiety levels could be found. However, Kolva et al. linked the influence that seems to be exerted by this variable to remaining survival time [13], which was not included in our analyses. In contrast to the findings of a previous study [28], we did not identify higher ratings of anxiety levels in specialized palliative care services than in other wards. Not surprisingly, medication with anxiolytics and antidepressants are predictors of anxiety and can also be found in the final multiple regression model described by Kolva [13].

Concerning medication, our calculations also indicate effects of antibiotic and cardiac usage. In particular, lower anxiety levels were reported in patients receiving cardiacs, whereas medication with antibiotics is likely to be accompanied by higher anxiety levels. This seems to be a suitable finding which proves a positive association between cardiac arrhythmias and high anxiety levels [9]. Those indications could be linked to the psychophysiological model of panic attacks as a vicious circle postulated by Pauli et al. [29]. In this study, differences in cardiac perceptions, anxiety levels related to those perceptions and heart period after those perceptions were investigated in patients with and without panic attacks. Although patients with panic attacks reported somewhat more cardiac perception than patients without any anxiety disorder, this difference was not significant. However, a highly significant difference could be found between these two groups regarding anxiety related to cardiac perceptions. Whereas in healthy controls cardiac perceptions did not elicit anxiety, subjects with panic attacks reported anxiety related to cardiac perceptions. Additionally, the study showed a clear positive association between levels of anxiety elicited by cardiac perception and faster heartbeat after those perceptions [29]. Since patients with a history of anxiety disorders may be more affected by cardiac perceptions, this suggests that those patients may benefit from medication with cardiacs in particular. For this reason, the assessment of psychiatric history could have a limited advantage. However, especially in patients with a shortened life span, the potential benefits must be balanced against the additional burden.

HOPE is already used as a quality management benchmarking system by many palliative care services in Germany [18–20]. Based on this assessment, we found a multivariable regression model to predict the risk of moderate or severe anxiety levels in terminally ill patients with a fair value. We therefore recommend supplementing clinical evaluation. The model can be integrated into existing digital documentation routines without much additional programming effort. It can facilitate early prediction of anxiety, which could be particularly important, for example, in the increasing efforts toward early integration of palliative care in the treatment of serious illnesses [30–32]. Above all, no additional effort is required from patients. This is consistent with requirements that diagnostic instruments, particularly for patients with terminal diseases, should be not very stressful and time-consuming [33, 34]. Further evaluations of the screening performance, possibilities to complement the model to enhance its predictive accuracy, and the benefits in clinical practice are advisable.

### Study limitations

Our results are based on a comfortably large sample size, but some limitations have to be taken into account when interpreting the results. The prevalence of anxiety in our population is very high compared to other findings [2]. This could be promoted for example by the given items in the tick boxes or perhaps it could be enhanced by an overestimation of anxiety levels by staff compared to self-assessments. However, on the other hand there are findings of similar or even higher prevalence of anxiety [35–37]. Therefore, it is doubtful whether there is an overrepresentation of anxiety in the present study. More importantly, due to the study design no statements about causalities are permitted. Even though we assumed different variables as possible predictors of anxiety, they were assessed simultaneously at the time of admission to palliative care service, and there could be a common underlying cause (such as disease progression) rather than a causal relationship between the variables. The vicious circle of anxiety and cardiac perception postulated by Pauli et al. [29] indicates a complex interrelation between physical symptoms and anxiety. Further research is needed here to explore causal and mutual or reciprocal influences.

HOPE, with its symptom and problem checklist, is a validated routine proxy assessment tool used by physicians, nurses and other healthcare personnel. When interpreting results it must be considered that patient reported outcome measures (PROM) would be the gold standard [38] and differences in clinicians’ and patients’ judgments of symptoms are evident [39–41]. However, due to weakness and/or cognitive impairment, for many patients in a palliative care situation it is impossible to use PROMs and thus a proxy assessment can safeguard data documented from all patients [42, 43]. There is evidence of satisfactory convergent validity for HOPE-SP-CL in investigations on patients’ self-assessment tools such as the MIDOS_2 or POS [21, 44]. In our service it is routine to use both.

Results are calculated only on the basis of data sets for which all variables have been completed. The proportion of missing values should be considered in the interpretation, particularly in the multivariable logistic model. No systematic errors were detected here. The reasons for missing data are unknown, and one hypothesis would be that missing data results because different occupational groups and team members bear responsibility for assessing the data. Additionally, no immediate beneficial effect results for healthcare personnel or patients, which could lead to a lack of motivation to complete the questionnaire conscientiously.

HOPE contains details on further psychological aspects such as feeling depressed, tension, and disorientation/confusion. Those psychological aspects were excluded from further analyses for two reasons. Firstly, the psychological items showed high intercorrelations. Secondly, those aspects as they are used in HOPE are not meant to be interpreted as psychiatric diagnoses. In particular, the variable feeling depressed, is not to be compared with the diagnosis of the mood disorder depression.

In addition, although we controlled for a wide range of variables there is no information about the influence of variables which are not included and by nature underrepresented in our analyses, such as a history of psychiatric disorders, acceptance of prognosis or religious beliefs [13, 15].

The comparability with other studies may be affected by the diversity of possible predictor variables determined by using HOPE. Furthermore, it must be considered that the present model has only a fair predictive value. Further research is needed to expand the range of and adapt possible predictor variables to develop models with higher predictive values and to further study interrelations with anxiety in patients in palliative care settings.

## Conclusions

The present study describes the development of a predictive model for anxiety in palliative care patients using data from the standard documentation routine in Germany. We conclude that this approach can improve the assessment of anxiety in patients in a palliative care situation.

This model could enhance the screening process but will not replace further clinical evaluations. It will be indispensable to perform individual interviews to provide appropriate support for anxious patients in palliative care settings, but this model could sensitize care providers for a plurality of interacting predictors and thus also improve early detection of possible risk factors of anxiety and anxiety itself.

## Supporting information

S1 Fig HOPE 2011 Basisbogen page 1. HOPE Basisbogen 2011 page 1.
(TIF)

S2 Fig HOPE 2011 Basisbogen page 2. HOPE Basisbogen 2011 page 2.
(TIF)

S3 Fig HOPE 2011 core data set. HOPE core data set questionnaire.
(TIF)

S1 File HOPE core data set 2007–2011.sav. Core data set of the study participants assessed with HOPE.
(SAV)
