# Supplementary figures and images for: Patients in palliative care—Development of a predictive model for anxiety using routine data
Source: PLoS One. 2017 Aug 3;12(8):e0179415. doi: 10.1371/journal.pone.0179415 (PMC5542653; doi:10.1371/journal.pone.0179415)

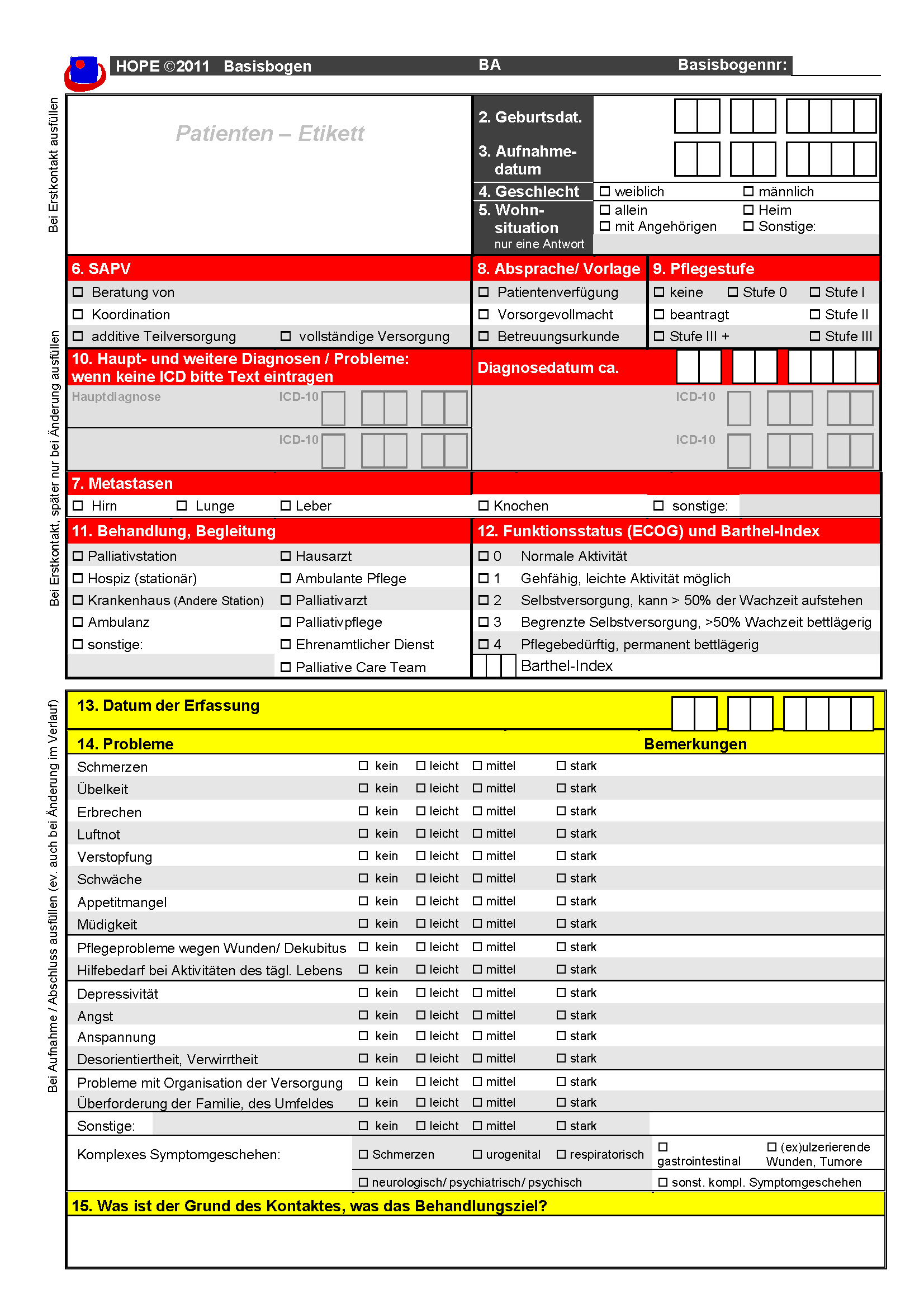

Supplement: S1 Fig — HOPE Basisbogen 2011 page 1. (TIF) [file pone.0179415.s001.tif]

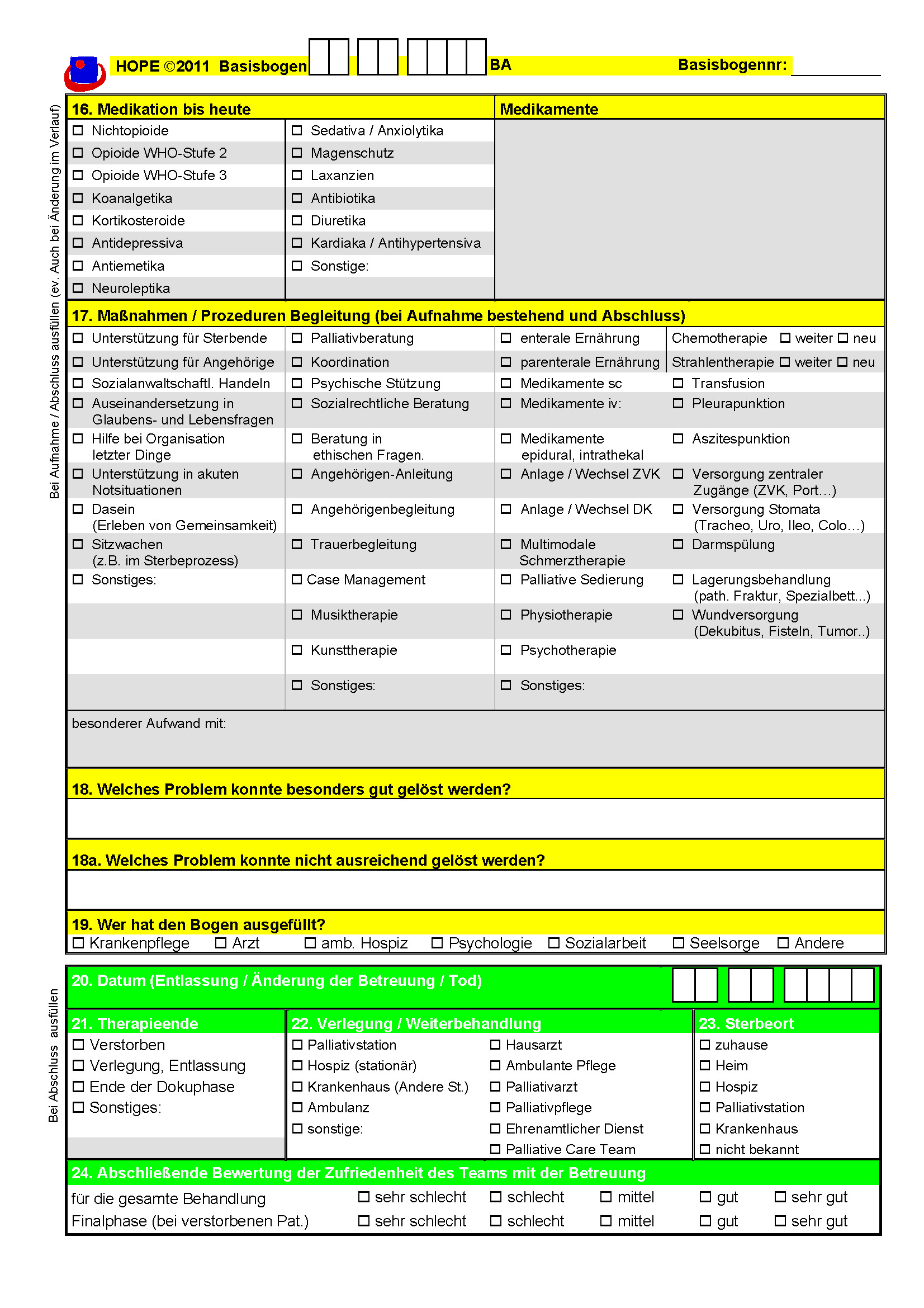

Supplement: S2 Fig — HOPE Basisbogen 2011 page 2. (TIF) [file pone.0179415.s002.tif]

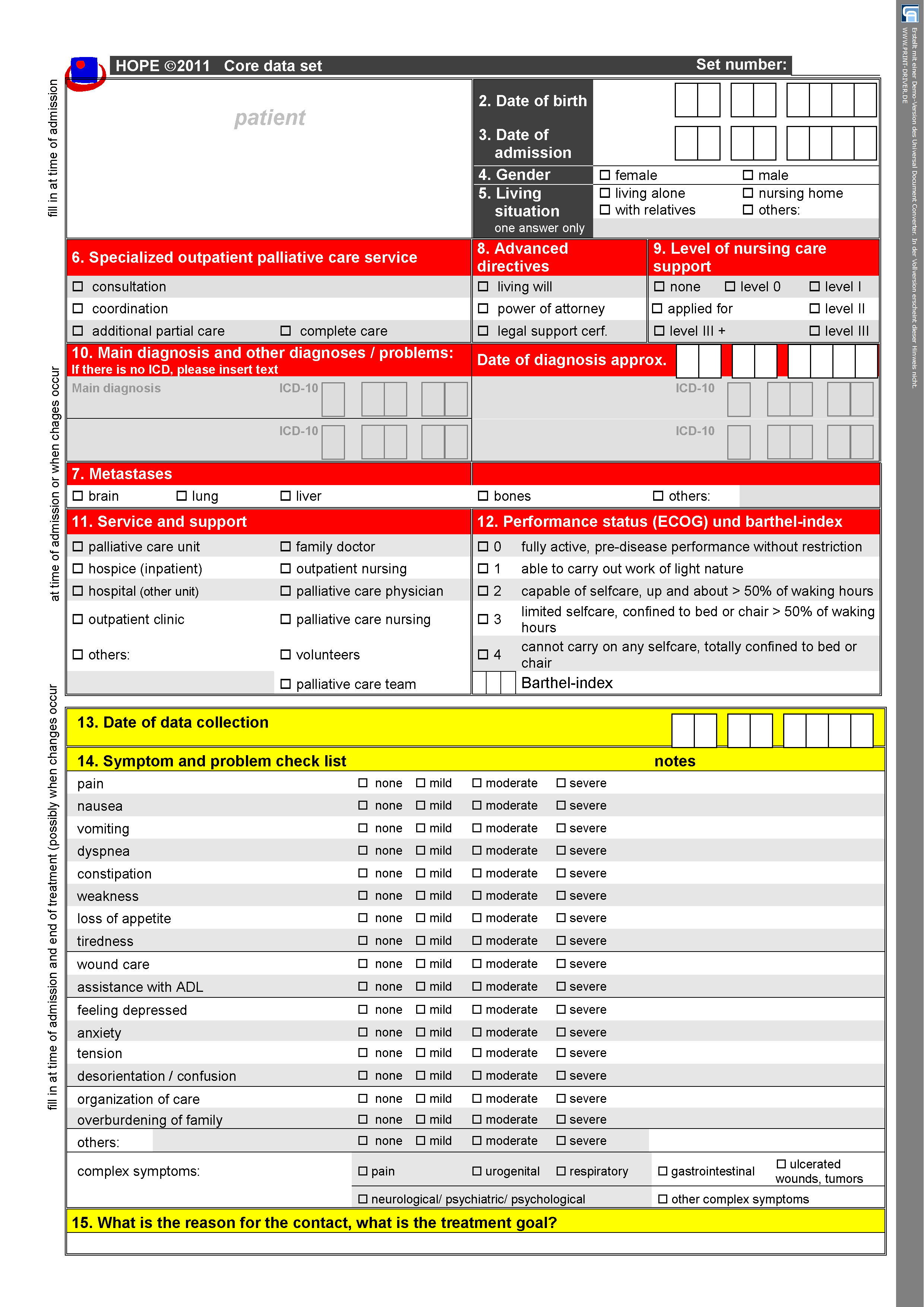

Supplement: S3 Fig — HOPE core data set questionnaire. (TIF) [file pone.0179415.s003.tif]
